# Homeopathy in the Michigan University

**Published:** 1875-09

**Authors:** 


### Editorial.

### Homeopathy in the Michigan University.

We copy from the *Detroit Review of Medicine and Pharmacy* the following correspondence, and also the extracts from the eighth anuual meeting of the American Medical Association, giving Prof. Palmer's views upon the subject of Homeopathy in the University. The creation of a College of Homeopathic Medicine in the University, with but two Professors, the other chairs being filled by the Professors of Anatomy, Physiology, Surgery, Chemistry and Obstetrics in the regular college, has attracted much attention from outside parties who have watched the movements of the old Faculty with considerable interest. As a College of Medicine cannot be complete without the above-named chairs, and as their lack is supplied by lectures in the regular college, it certainly seems that the members of the old faculty are also members of the Homeopathic school. The new rule by which the faculties of neither school sign the diplomas, excuses the old faculty from placing their sanction upon the homeopathic graduates, but as they are examined by the Professors of Anatomy, Physiology, Obstetries, etc., it seems that this is somewhat after the manner of a mere technical dodge to avoid appearing to do what they really do. Withholding further comments, we append the letters and extracts :

ANN ARBOR, August 17, 1875.

*Editors Detroit Review of Medicine and Pharmacy:*

GENTS—Believing that the communication of Prof E. S. Dunster, in reply to the article in the *Medical Record*, in reference to the history and relation of homeopathy to the old school in the medical department of the University, contains some elements of error calculated to mislead the minds of your readers, I beg to submit another view, presenting what is believed to be a true exhibit of the degree of union or fusion of the faculties of the two colleges now established in the medical department.

The Board of Regents, in adopting the resolution "That a homeopathic college be established," of course implied that a definite curriculum of studies should be pursued to qualify the student for the honor of the degree of doctor of homeopathic medicine. The curriculum, it was also enacted, should be equal to that required by the student of the regular school in the same department. In conformity with established usage, this degree could be conferred by the Regents only on the recommendation of the faculty of the college of homeopathy. Now, as the faculty of any college consists of the teachers especially employed to qualify and recommend the student for the degree, it will at once be seen that the professors of anatomy, of physiology, surgery, chemistry and obstetrics, must be united practically with the newly-appointed professors of homeopathic practice and materia medica to constitute such a faculty. The merely technical dodge that they are by appointment members of another faculty, will not convince any one to the contrary in opposition to this single prominent practical fact. The professors of the old school, therefore, bear the same relation as teachers, examiners, and final judges of qualifications for the degree in homeopathy, as they do to the class of candidates for honors in the school of regular medicine. By another technical dodge, to avoid an humiliating self-conviction by the faculty of the old school, the Regents have abolished the time-honored custom of affixing the signatures of the faculty to the diplomas. Hence, in the future the faculty no longer testify to the qualification of the graduate, but only the president and secretary.

I think, therefore, it will be conceded by every one who prefers actual facts to mere subterfuges, that as practically more than two-thirds of the faculty of the so.called college of homeopathy are also members of the faculty of the college of regular medicine, that the fusion of the faculties of the department of medicine is nearly complete ; and, further, that of whatever of merit or obloquy may, in the estimation of the regular profession, attach to such union for the promulgation of the absurd dogmas of homeopathy, the faculty of the old school will be entitied to a full share.

Very respectfully, A. S.

DETROIT, August, 1875.

*Editors of Detroit Review of Medicine and Pharmacy:*

I have this moment laid down a Philadelphia *Medical and Surgical Reporter* of August 28, 1868, and under the head of "Correspondence" I find a letter from Prof. A. B. Palmer, of our State University, explaining the position of the medi cal department of that institution in relation to the appointment of a homeopathic professor, which was unsuccessfully attempted at that time.

Stating that the Regents under no circumstances would establish a chair of homeopathy in the department at Ann Arbor, "*to disturb its curriculum and introduce confusion and ruin*," he finishes up in the following style: "It can readily be understood that the medical faculty have been placed in a very embarrassing position; the pressing wants of the University, and charges of illiberality, unfairness and prejudice being on the one hand, and professional honor and interests of medical science on the other.

' Hastily abandoning their positions would have led to the triumph of folly and error, and the ruin of the largest, and, as they believe, one of the best medical schools in the country; whilst quietly yielding to such an unnatural association would have been a sacrifice of self-respect and professional reputation which could not for a moment be thought of They have done what they have regarded their duty to the profession and themselves and we are happy in the belief that they have not sacrificed the honor or interests of either,

" The most impressive lessons of wisdom are bought by experience, and if the lesson of these experiences be heeded, no further attempts will be made to mingle in the same institution elements so totally incompatible as scientific medicine and the exclusive and absurd system of homeopathy."

I have called your attention to the foregoing simply with the view of asking Prof. Palmer, as one who years ago sat under his teachings, and therefore could not fail to imbibe many of the ideas he held regarding homeopathy at that time, and which he must still have held when he penned the above, how he reconciles his conscience to the present position of things in the University? Are "professional honor and the interests of medical science" less dear to him now than then? Has he concluded to sacrifice self-respect and professional reputation by" quietly yielding to "such an unnatural association?" Or has he just discovered that the "elements so totally incompatible as scientific medicine and the exclusive and absurd system of homeopathy" are more compatible than he thought they were? I pause for a reply. Verily, consistency is a jewel!

HENRY A. CLELAND, M. D.

PROF. A. B. PALMER'S VIEWS—The friends of this gentleman have waited long and patiently for an expression of his opinions concerning his present relations. They are interested to know whether he has changed his principles on which for so long a time and so valiantly he fought the enemies of scientific medicine. Upon what platform does he now stand? We cannot answer this question, but we can show what he once believed, This we shall do by some brief extracts from the proceedings of the eighth annual meeting of the American Medical Association.—*Peninsular Journal of Medicine*, Vol. 3, p. 21.

Dr. Atlee, of Pa., offered the following resolutions:

*Resolved*, That, to secure efficient teaching in medical schools where a prime object is to enforce practical precepts, a large degree of harmony must exist among the teachers, and confidence be reposed in them by their pupils.

*Resolved*, That any such unnatural union as the mingling of an exclusive system, such as homœopathy, with scientific medication in a school, setting aside all questions of its untruthfulness, cannot fail, by the destruction of union and confidence, and the production of confusion and disorder, unsettling and distracting the minds of the learners, to so far impair the usefulness of teaching as to render any school adopting such a policy unworthy the support of the profession.

The resolutions were seconded, accompanied by the remark that they had reference to an attempt made in Michigan to thrust homœopathy into the medical department of the University of that State. Dr. Palmer, the delegate from the medical department of the University, was called upon for a statement upon the subject. We quote but a small portion of his somewhat extended remarks.

He said that such an attempt had been made, and that a report had gone forth that such a chair had been established. An act had passed the Legislature during the last hours of its recent session providing that a chair of homœopathy should be established in the medical department of the University, although the regents had not, and he believed would not, make any such appointment.

He had no doubt that the sentiments contained in the resolutions offered by the gentleman from Pennsylvania were those of this learned and dignified body—a body which had been spoken of as "the assembled wisdom of the profession," and that all men of sense and principle in the profession, who had reflected upon the subject, or who knew anything of medical schools, entertained such views.

He well knew that a school adopting such a course as was indicated and denounced in the resolutions would fall under the deep condemnation of all regular physicians, and that without their confidence and support it could be supplied only by such materials as the irregular are usually made of and must fail and become extinct as a respectable and orthordox institution. It could not and would not receive your support. This being the unquestioned fact, the sentiments of the resolutions being such as the profession entertained and would act upon, he could but approve whatever might be the result of their expression at this time and by this body. If there were any present that entertained other views; if there were any that would extend their patronage to a school where such incongruities were brought together; where the lecture of one hour was flatly contradicted by that of the next; where all union and all harmony, and all concord were destroyed; where a policy was adopted which would be the same in effect as the introduction of a Mohammedan teacher in a Christian Sunday school or bible class; if any could approve or tolerate such a course, he hoped we should hear from them; but if, as he anticipated, we were all of one heart and one mind, let there be a united expression, which should show to the regents of the University of Michigan, what would be the result to their cherished institution.

Again, the founders of the medical department of the University sought in its organization to follow your directions; those connected with it now seek to render it worthy of your approval; and in the worst event, should they fail to accomplish this object, they intend at least to save their own professional honor untarnished amid the ruin. The resolutions of Dr. Atlee, and the remarks upon them were received with strong expressions of approval, and the resolutions were unanimously adopted."

Since the above was written we have received a "Statement" from the Faculty of Medicine and Surgery in the Michigan Uuiversity concerning their relations to

Homeopathy in the University, signed by Dr. A. B. Palmer in behalf of the Faculty.

This statment does not throw any light upon the subject, but it takes a stand decidedly in opposition to that assumed by Prof. Palmer in his remarks, already quoted, at the American Medical Association.

If Homeopathy is to be taught at the Michigan University let it be taught by a complete faculty which shall in no wise be composed, in part, of the members of the present Faculty of Medicine and Surgery. Until such is the case the profession must look with a degree of suspicion upon the present state of affairs, and students attending the Medical Department of Michigan University will be regarded with a degree of suspicion by their professional Associates. It will be interesting to see whether in the Announcement of the Homeopathic lectures the Professors in the regular department will be included under the head of faculty, with the Homeopathic Professors of Materia Medica and of Theory and Practice of Medicine.

